# Supplementary material for: Linguistic correlates of societal variation: A quantitative analysis
Source: PLoS One. 2024 Apr 16;19(4):e0300838. doi: 10.1371/journal.pone.0300838 (PMC11020942; doi:10.1371/journal.pone.0300838)
Supplement: Attachment — Submitted filename: Response to reviewers.docx [file pone.0300838.s001.docx]

**Reply to reviewers**

Reviewer #1: **General assessment**

The study presents the results of multiple analyses of linguistic and societal variables to establish whether the correlation between them exists and to infer if the alleged trade-off between morphological and syntactic complexity is influenced by these correlations. For this purpose, a wide range of societal variables was obtained from different databases, and the features from WALS pertaining to morphological and syntactic complexity were selected. The authors go to great lengths to overcome the Galton’s problem by rerunning the the analyses and checking if the results are supported when family relatedness and regional proximity are accounted for. The presentation of the findings and the language in general are clear and easy to follow. The authors also offer the novel interpretation of the findings in the light of the prevalence of procedural or declarative memory. Overall, I recommend the paper for publication after addressing the major concerns with 1) the presentation and interpretation of results, 2) explaining criteria underlying the distinctions between morphological and syntactic complexity and principles for including the individual WALS parameters, and 3) data availability. I also provide minor comments related to literature review and citations.

Answer: We thank the reviewer for his/her insightful reading of the manuscript and for his/her recommendations. We have extensively revised and improved the paper, and we think we have addressed these major concerns, as well as the minor issues listed below. Regarding 1) we have reanalyzed the data using a different statistical approach and generated new graphs of the results. Concerning 2) we have clarified the criterion for classifying features as morphological or syntactic (in essence, we adopted the simple rule that if a feature classification is related to grammatical rules within a word, then it is considered as a morphological classification; in addition, if a feature classification relates to grammatical rules between words, then we consider it as a syntactic classification). Also, instead of merely classifying the features of interest after conducting the analyses, we have now classified all of them before performing the analyses. We expect this to result in a more rigorous testing, particularly, regarding the hypothesis of a correlation between complex morphology (as a whole) and sociopolitical esotericity, and between complex syntax (as a whole) and exotericity. Finally, with regards to 3) we now provide a link to a GitHub repository (<https://github.com/cshnican/XSlanguages>) which contains all our raw data and codes. All the changes we have made in the previous version of the manuscript are highlighted in gray.

**Major comments**

**Results and their interpretation**

If my understanding is correct, only 10 tested relationships (out of 844) have survived both controlling for family relatedness and region proximity and emerged as significant (at least at 0.05 level), i.e. there are 10 cells with FR*, FR**, or FR***. These are the correlations that should be described in the results section (rather than those that additionally appear significant(**,***,***) but disappear after family or region are accounted for). While the authors make it clear which relationships hold after the additional analyses and which do not, the spurious correlations (the cells that do not contain FR*, FR**, and FR***) should not be given as much attention or interpreted as significant and showing “a clear trending in the direction predicted” (p. 15).

Answer: We have now adopted this criterion systematically, so we make clear which relationships are significant, and which ones survive after controlling for both geography and phylogeny. In the Discussion, we focus on the latter.

p. 14. “Most of the results reported in sections 3.1. and 3.2. survive controlling for family relatedness, but not all. We interpret this to mean that the correlations that do not survive can be attributed either to family relatedness or to direct correlations between societal and linguistic factors. This can also mean that both factors are contributing to the attested correlations, and that more discerning tools for testing will be needed to disentangle one 15 factor from the other. On the other hand, only very few correlations in our results survive controlling for regional factors.”

If there were direct correlations between societal and linguistic factors, the correlations would have survived controlling for family relatedness and regional proximity. The idea behind those additional tests addressing the Galton’s problem is checking whether the initial correlations hold when confounding factors are taken into account. If the correlations disappear in the analyses incorporating control for family relatedness and/or regional proximity, then these results are spurious. If the correlation remains when the effects of family and region are included in the analyses, these results cannot be explained away merely by non-independence between languages.

Answer: this part has been removed from the revised manuscript. As noted, in our Discussion we now focus on the correlations that survived after controlling for both factors.

Additionally, I recommend to accompany the supported relationships in the results section not only with the direction of the correlation (positive/negative), but also with specifications of the strength of the correlation to clarify which relationships are weak/moderate/strong (based on the Figure 1 and the presented the correlation coefficients).

Answer: thank you for your suggestions. In the revised manuscript (Figures 2 and 3), we now show the 95% confidence interval / credible interval of the regression coefficient of each test we did. A higher absolute value of the regression slope indicates a stronger effect.

**Morphological and syntactic complexity**

The authors provide the principles based on which features pertaining to morphological complexity are included. e.g. p. 6 “We then say that the latter category is more complex than the former one, following the principle that the more symbols needed to fully describe a grammatical rule, the more complex the rule is (Li & Vitanyi, 2008). In this case, more text is needed to describe a grammar with affixes than to describe a grammar without them, since to describe the former, one needs to specify explicitly the forms, functions and locations of the affixes, whereas no description is needed for the latter.”

Do the same principles apply when syntactic complexity parameters are chosen? Or are only the criteria based on the syntactic theory (provided in the discussion) are used instead? It would be useful to provide the basic criteria for inclusion the features to the syntactic complexity metric early on.

Answer: the same criterion applies to syntactic complexity

If my understanding is correct, the selection of features on the dominant word order imply that dominant word order is treated as more complex. However, if the rules criteria is applied, a language with no dominant word order could count as more complex as more rules could be required to describe the conditions under which different orders are used. Please clarify.

Answer: We treat a dominant word order as involving a rule in syntax, while free word orders may involve more complexity in other domains, such as semantics or pragmatics. This is how we explain it in the paper:

“By contrast, our results for syntax are consistent with the hypothesis that Type X languages are characterized by more syntactic complexity, specifically with more syntactic layering, as well as with more obligatory syntactic categories and distinctions. In particular, among the strongest findings, we observed the existence of dominant word order and obligatory pronouns in subject positions. Within the minimalist program (see e.g. Adger, 2003), both relate to the syntactic layer of Tense Phrase (TP). Following this theoretical framework, the category Tense, the head of the TP, has a strong feature in some languages, requiring the specifier of TP (the subject position) to be filled, whether by moving a noun phrase from a lower layer into it, or by inserting a meaningless pronoun in this position, as found in e.g. *It is snowing* in English. This rigid rule of syntax contributes to both a dominant word order (with the subject position rigidly in the specifier of TP), and to the obligatory use of pronouns in the subject position.

Furthermore, how exactly are the distinctions made between features belonging to either morphological or syntactic complexity sets? For instance, why is the nominal plurality feature is the part of syntactic complexity, whereas future tense is classified as morphological complexity (if both features can be marked inflectionally)?

Answer: We acknowledge this is a key question and in fact, an active debate in linguistics. As we now clarify in the revised Methods, we have adopted a simple criterion: if a feature classification is related to grammatical rules within a word, then it is considered as a morphological classification; in addition, if a feature classification relates to grammatical rules between words, then we consider it as a syntactic classification. In the Methods section, we also now provide examples that should help the reader understand how we have applied this criterion. That said, it is clear that some features can be assigned to these two domains, as grammatical cases: they entail making changes in the word form, but they fulfill a role at the sentence level too. This is why in our new analysis we have adopted, specifically, a quadripartite criterion, so we distinguish between purely morphological features (M), purely syntactic features (S), features pertaining to both domains, but predominantly related to morphology (Ms) and features pertaining to both domains, but predominantly related to syntax (mS).

Please also clarify whether the inclusion of Accusative alignment as a parameter in syntactic complexity implies that e.g. Ergative alignment would be “less complex”.

Answer: this is no longer discussed in the revised manuscript

**Data availability**

Based on the columns in the attached .csv file, the supplementary materials contain only data from Glottolog (columns on sampled languages, their location, family, etc). However, the values for WALS variables along with variables from DPlace and Ethnologue are not provided, which prevents the interested readers from replicating the study.

Answer: All the missing variables can be now found in the GitHub repository

Furthermore, I suggest making the code used for running the analyses, data wrangling and generating the main results figure (Figure 1) available.

Answer: All this can be now found in the GitHub repository

While based on the description of the methods, the statistical analysis appear to have been performed appropriately and rigorously, the absence of the code (for analyses) currently makes it impossible conclude whether it is the case, so I highly recommend making the code available.

Answer: we hope this has been properly fixed now that our data and codes are available in the GitHub repository and that we have improved the description of our methodology for the statistical analysis.

**Minor comments**

p. 3 “Increasing evidence suggests, however, that overall language complexity might differ cross- linguistically (e.g., Sampson, Gil, & Trudgill, 2009; McWhorter, 2011; Koplenig, Wolfer, & Meyer, 2022).” This implies that the trade-off hypothesis is not taken for granted and many studies reveal cross-linguistic correlation rather than an expected negative correlation, but I did not encounter any explicit claims about the absence of (overarching) trade-offs that would reference the studies that find no evidence for trade-offs (examples could be Shosted (2006), Sinnemäki (2008), Miestamo (2009)).

At the same time, since the paper consistently mentions the trade-off between morphology and syntax, it would be helpful to cite studies that suggest (and ideally test) this specific trade-off.

Answer: We have revised this part as per suggested. We now distinguish between the issue of complexity and the issue of trade-offs. We cite the papers mentioned by the reviewer and also a recent paper also addressing this issue (Benítez-Burraco et al., 2024).

p. 3. “Moreover, evidence also suggests that language features and the trade-offs between subparts of language grammars can be impacted by extralinguistic factors.” The works cited after this sentence involve the examination of the relationships between individual features or aggregated scores and societal features, but none of them explore how “the trade-offs between the subparts of language grammars” are influenced by extralinguistic factors. In fact, few works address how the trade-offs can be impacted by such factors. Sinnemäki (2020) could be treated as such a study, which is not cited (though Sinnemäki 2009, which is cited, explores an adjacent question).

Answer: we now acknowledge this limitation, cite the paper by Sinnemäki (2020), and highlight the novelty of our approach, since we do address this potential effect of trade-offs specifically.

p. 3. Please briefly spell out how exactly vegetation has an influence on sound inventories (the way it is done for tonal languages and dry climates right after the following sentence): “A familiar example is the effect of vegetation on sound inventories (Maddieson and Coupé, 2015), which parallels what has been observed in many vertebrates (Boncoraglio and Saino, 2007; Ey and Fischer, 2009)”.

Answer: this has been fixed

p. 4. I suggest citing appropriate literature to support the claims made in this sentence: “Recent typological surveys suggest that the number of speakers, the degree of bilingualism, the tightness or the looseness of the social networks, the sociopolitical organization, or the number of adult learners of a language correlate, and perhaps explain, the types of morphology or syntax exhibited by the world languages”.

Answer: this has been fixed

p. 4. Since Lupyan & Dale (2010) do not refer to their metric scores as “the index of agglutination”, but use “morphological complexity measure/score”, this term might be more appropriate.

Answer: this has been fixed

p. 4. Replacing or explaining the “hypotaxis” term at first usage will make the sentence more transparent to non-specialists. Regarding the same term, it is not clear whether it is used in the same meaning as “(syntactic) layering” or if these are two different concepts: see p. 14 "a language with more layering and more hypotaxis”, p. 15 “syntactic layering (hypotaxis)”, p. 16 “syntactic layering and hypotaxis”

Answer: after checking some of the literature about hypotaxis (particularly, Sinnemäki’s “On Halliday’s distinction between embedded and hypotactic clauses”), we decided to use “embedding” instead of “hypotaxis” throughout the whole paper, as the former is a commoner term in the literature which needs no further clarification.

p. 4. Please clarify whether the distinction between Type A and Type B languages follows Kusters (2003) where a very similar distinction is made.

Answer: Our distinction (now Type S vs Type X) is indeed similar to Kusters’ classification of the languages spoken by Type 1 and Type 2 speech communities. We now acknowledge this in the revised manuscript

p. 8. The inclusion of the “permission of cousin marriages” variable needs to be motivated given that this variable has never been used or mentioned as a potential variable in the previous studies. ****

Answer: in our new analyses, this feature behaved quite dissimilarly to the others, and we decided to exclude it from our research, also by the reason mentioned by the reviewer (= it precludes any comparison with previous work)

p. 9. “We consider that a correlation exists between a linguistic feature and a societal factor even if just one of the societal parameters shows such a correlation, but clearly the correlation should be considered stronger if multiple societal parameters correlate with a linguistic parameter.” Since such a diverse set of societal variables results in different language samples on which individual pairwise correlations are tested, the correlation should not be considered stronger or weaker based on how many societal parameters are correlated with linguistic variables.

Answer: In the revised version of the manuscript, we first removed the societal features related to cousin marriages, since there is no existing theory suggesting a relation between such features and exotericity/esotericity. Then, we collapsed the remaining features into one single metric by performing a principal component analysis. As a result, there is now only one test for each grammatical classification instead of 12, and now we say there exists a correlation between sociopolitical complexity and a grammatical classification if the statistical test comes out to be significant.

**Citations and references**

p. 13 “(e.g., Bošković, 2008, and much subsequent work).” and p. 15 “(e.g., Lyons 1999, and subsequent work)”. Please specify the subsequent work.

Answer: this part was removed from the revised manuscript

p. 17. "In their own words, they test the hypothesis “that languages in highly exoteric societies have (1) less phonologically fused grammatical markers (fusion) and (2) overall fewer obligatory explicit markers (informativity) compared to languages in low-exotericity societies.” Please provide the page for the direct quote.

Answer: this was not actually a literal quotation, so we have removed the quotation marks

Make sure that the references of the journal articles are formatted uniformly. For instance, the journal name in Ullman (2004) and some other references is not in italics.

Answer: we have checked (and revised) this throughout the whole list of references

**References**

Kusters, Wouter. 2003. *Linguistic complexity*. Netherlands Graduate School of Linguistics.

Miestamo, M. (2009). Implicational hierarchies and grammatical complexity. In Sampson Geoffrey, David Gil & Peter Trudgill (eds.), Language complexity as an evolving variable, 80–97. Oxford: Oxford University Press.

Sinnemäki, Kaius. 2020. Linguistic system and sociolinguistic environment as competing factors in linguistic variation: A typological approach. *Journal of Historical Sociolinguistics* 6(2). 20191010. https://doi.org/doi:10.1515/jhsl-2019-1010.

Sinnemäki, Kaius. 2008. Complexity trade-offs in core argument marking. In Matti Miestamo, Kaius Sinnemäki & Fred Karlsson (eds.), *Language complexity: Typology, contact, change*, 67–88. Amsterdam/Philadelphia: John Benjamins.

Shosted, Ryan K. 2006. Correlating complexity: A typological approach. *Linguistic Typology* 10(1). 1–40.

Reviewer #2: Despite my skepticism (which I will explain below), this work should definitely be published (with minor revisions). It is well written, thought-provoking, and brings together systematic comparable data from different disciplines (linguistics, anthropology, neuropsychology), where linguistic and societal data come from large published databases. Such work should be encouraged and will certainly be a stimulus for future interdisciplinary research.

Answer: We thank the reviewer for his/her insightful reading of the manuscript and for his/her positive comments and overall recommendation

My basic problem with the article is that, in my opinion, the data presented do not sufficiently support the main hypothesis, which is: "languages spoken by exoteric societies tend towards more complex syntaxes, while languages spoken by esoteric societies tend towards more complex morphologies." (from the abstract).

Even if one accepts that syntax and morphology can be separated as clearly as the authors seem to assume (which I don't think is the case), the statistical tests would have to yield different results to confirm the initial hypothesis. If I understand the authors' method correctly, they are trying to correlate linguistic features related to morphological and syntactic complexity with socio-demographic features. If correlations emerge, the important question remains whether these correlations can also be explained by genealogical relationship ("Family") and/or by geographical proximity ("Region"). This means that all potential linguistic features must first survive controlling for family AND region before they can be interpreted as evidence for the main hypothesis above.

It looks like the authors have a completely different take on this. Because according to their quantitative evaluation, there are only very few linguistic features in Fig 1 that show a significant correlation (marked with an asterisk) and at the same time survive controlling for family and area (FR) (for instance D. Possessive Classification; E. Presence of Action Nominal Constructions; G. Use of Relative Pronouns on Subjects and Use of Relative Pronouns on Obliques: BTW, I doubt the accuracy of this last result given the clear areal distribution of feature values in the two relativization features).

And yet the text of the article pretends over and over that this lack of evidence is not a lack of evidence at all. Instead, it is said that the many linguistic features that can be explained by family and/or region are also good arguments for the hypothesis cited above. See for example this quote: "Most of the results reported in sections 3.1. and 3.2. survive controlling for family relatedness, but not all. We interpret this to mean that the correlations that do not survive can be attributed either to family relatedness or to direct correlations between societal and linguistic factors.” (p14) But then the authors admit: "On the other hand, only very few correlations in our results survive controlling for regional factors.” (15) But nothing follows from this in their argument. Instead the authors conclude: "As reported in the Results section, our results align with our broad hypothesis.” (p16) This kind of reasoning is somehow unsatisfying for me. I would expect a revision to address this mismatch more clearly.

Answer: We agree with the reviewer’s concerns, and we have tried to fix them in the revised manuscript. All the changes we have made in the previous version of the manuscript are highlighted in gray.

First, with regards to the syntax vs morphology divide, we acknowledge this is a key question and in fact, an active debate in linguistics. As we now clarify in the revised Methods, we have adopted a simple criterion: if a feature classification is related to grammatical rules within a word, then it is considered as a morphological classification; in addition, if a feature classification relates to grammatical rules between words, then we consider it as a syntactic classification. In the Methods section, we also now provide examples that should help the reader understand how we have applied this criterion. That said, it is clear that some features can be assigned to these two domains, as grammatical cases: they entail making changes in the word form, but they fulfill a role at the sentence level too. This is why in our new analysis we have adopted, specifically, a quadripartite criterion, so we distinguish between purely morphological features (M), purely syntactic features (S), features pertaining to both domains, but predominantly related to morphology (Ms) and features pertaining to both domains, but predominantly related to syntax (mS).

Second, with regards to Galton’s problem and the control for geography and phylogeny, in the Discussion we now focus on the correlations that survive after controlling for both factors. Even more importantly, we have adopted a new statistical method. This method is essentially a Bayesian mixed-effects regression, but what makes it different from other mixed-effects regressions commonly used in the literature is that here the random effect structures are exactly specified. Following Shcherbakova et al. (2023), we had a random effect for language relatedness and a random effect for geographical proximity, by specifying two correlation matrices, one for each random effect: the first correlation matrix stipulates that languages that are genetically more similar (calculated from the global EDGE tree, Bouckaert et al., 2022) to each other correlate more, and the second matrix stipulates that languages that are spoken by societies closer to each other (calculated from the coordinates given in the WALS database) correlate more. Compared to our previous method, this method does a better job stripping away the effect of language relatedness and language contact, and the effect found from this analysis can serve as a more compelling piece of evidence.

Finally, in spite of these improvements, the limitation raised by the reviewer unfortunately still holds: ideally all features related to morphology and syntax should be significant if we want to make strong claims about the effect of sociopolitical complexity on language complexity. Accordingly, in the revised version of the manuscript, we acknowledge this limitation of our approach, tone down our claims, and focus on specific correlations we find of interest for current discussions on the topic addressed by our study.

.”

The declarative/procedural memory discussion should be relegated to the background or omitted altogether, because nothing is provided except the formulation of predictions: this is too weak to be published in this form. A systematic cross-linguistic data collection on formulaic, irregular language use would probably be the first step to do. But conceptually, such an endeavor is extremely tricky, since we know of vast numbers of formulaic constructions at all levels and domains of grammar in all sorts of language varieties. I wonder what the comparative concept of "formulaic use" might be, and how one might obtain meaningful data without working with language corpora (and frequencies of types/tokens). Given this complex context, it is far from clear how the projected testing of the hypothesis that different memory types are associated with different language types can be performed using "standard cognitive experiments" (p19).

Answer: we agree with the reviewer. Accordingly, we have removed from the paper the references to the memory issues.

Accordingly, the title of the paper should be changed because no empirical research is presented on structural linguistic features and a possible correlation with the two types of memory. This article is *only* about potential correlations between linguistic and societal features. This should also be reflected in the title.

Answer: we also agree on this and have fixed this issue as per suggested.

Please provide the statistical data as well as the data on feature classification (plus the 94 WALS features, which are only retrievable from Fig a) in the supplementary data (is it there? I haven't found it). Please also highlight *which* of the societal features are involved in which positive/negative correlations. As far as I can see, this information is not given systematically, with the exception of Figure 1, where everything is squeezed in but not easily retrievable by the reader.

Answer: All our raw data and codes can be now found in the GitHub repository (https://github.com/cshnican/XSlanguages). The positive/negative correlations should now be clearly identifiable in both the text and the figures.
